# Biomimetic one-pot synthesis of gold nanoclusters/nanoparticles for targeted tumor cellular dual-modality imaging

**DOI:** 10.1186/1556-276X-8-170

**Published:** 2013-04-15

**Authors:** Jing Lin, Zhijun Zhou, Zhiming Li, Chunlei Zhang, Xiansong Wang, Kan Wang, Guo Gao, Peng Huang, Daxiang Cui

**Affiliations:** 1 Key Laboratory for Thin Film and Microfabrication of Ministry of Education, Institute of Micro-Nano Science and Technology, Shanghai Jiao Tong University, 800 Dongchuan Road, Shanghai 200240, People’s Republic of China; 2 Institute of Dermatology and Department of Dermatology at No. 1 Hospital, Wenzhou Medical College, Wenzhou 325000, People’s Republic of China

**Keywords:** Bovine serum albumin, Gold nanocluster, Gold nanoparticle, Folic acid, Cellular imaging

## Abstract

Biomimetic synthesis has become a promising green pathway to prepare nanomaterials. In this study, bovine serum albumin (BSA)-conjugated gold nanoclusters/nanoparticles were successfully synthesized in water at room temperature by a protein-directed, solution-phase, green synthetic method. The synthesized BSA-Au nanocomplexes have fluorescence emission (588 nm) of gold nanoclusters and surface plasmon resonance of gold nanoparticles. The BSA-Au nanocomplexes display non-cytotoxicity and excellent biocompatibility on MGC803 gastric cancer cells. After conjugation of folic acid molecules, the obtained BSA-Au nanocomplexes showed highly selective targeting for MGC803 cells and dual-modality dark-field and fluorescence imaging.

## Background

Fluorescent materials as prominent tools have been widely investigated and applied in many practical fields, including mineralogy, gemology, chemical sensors (fluorescence spectroscopy), fluorescent labeling, dyes, biological detectors, and, most commonly, fluorescent lamps [1-3]. To date, there are three main types of fluorescent materials: organic dyes, fluorescent proteins, and nanotech probes [4]. Compared with existing organic dyes and fluorescent proteins, nanotech probes can offer signals that are several folds brighter and hundreds of times more stable [5,6]. The range of substances of nanotech probes mainly includes carbon, semiconductors, and precious metals [4].

Carbon nanotubes, due to their natural photoluminescence in the tissue-penetrating near-infrared region, have been successfully explored as potential imaging tools [7]. Recently, carbon dots as a relative newcomer have multicolor emission capabilities and non-toxic nature, which enable them to be engaged in a wide range of applications in the biomedical field [8]. Unlike semiconductor nanomaterials or quantum dots (QDs), however, the fluorescent properties of carbon-based probes are harder to control [4]. QDs (such as CdSe, CdTe, and PbTe) have received broad attention due to their unique optical and biochemical features. However, the release of Cd^2+^, Pb^2+^, or other heavy metal ions arouses cytotoxicity and is a potential environmental hazard, which limits the applications of QDs [9,10].

More recently, precious metal nanoparticles (such as gold nanoclusters (AuNCs)) are highly attractive because of their high fluorescence, good photostability, non-toxicity, excellent biocompatibility, and solubility [11,12]. Biomimetic synthesis has become a promising green pathway to prepare nanomaterials [13-16]. Ying’s group used the protein bovine serum albumin (BSA) as a scaffold to make AuNCs (<1 nm) with red emission (640 nm) via a simple, one-pot, solution-phase, green synthetic route within 12 h [17,18]. Zhu et al. have successfully prepared AuNCs with near-infrared emission and Au@AgNCs with yellow emission using a BSA-assisted sonochemical approach [19]. Therefore, organic fusion of the fluorescence emission of AuNCs and the surface plasmon resonance of gold nanoparticles (AuNPs) enables dual-modality dark-field and fluorescence imaging.

Herein, we reported a simple ‘one-pot’ synthesis of gold nanoclusters/nanoparticles by using chloroauric acid (HAuCl_4_·3H_2_O) along with hydrazine monohydrate (N_2_H_4_·H_2_O) as reducer in the presence of BSA under vigorous stirring. The synthesized AuNCs and AuNPs own fluorescence emission (588 nm) and surface plasmon resonance (500~700 nm), respectively. The BSA-Au nanocomplexes display non-cytotoxicity and excellent biocompatibility on MGC803 gastric cancer cells. After being conjugated with folic acid molecules, the BSA-Au nanocomplexes demonstrate various functions such as tumor targeting and dual-modality imaging.

## Methods

In a typical experiment, aqueous HAuCl_4_ solution (5 mL, 50 mM) was added to BSA solution (10 mL, 3 mg/mL) with vigorous magnetic stirring at room temperature. Afterward, the mixed solution was vacuumized and kept static under nitrogen protection for 2 h. Then, 0.2 mL of N_2_H_4_·H_2_O was injected into the vacuumed solution under magnetic stirring. After reaction, the resulting mixed solution was aged under ambient conditions for 24 h.

## Results and discussion

**Figure 1:**
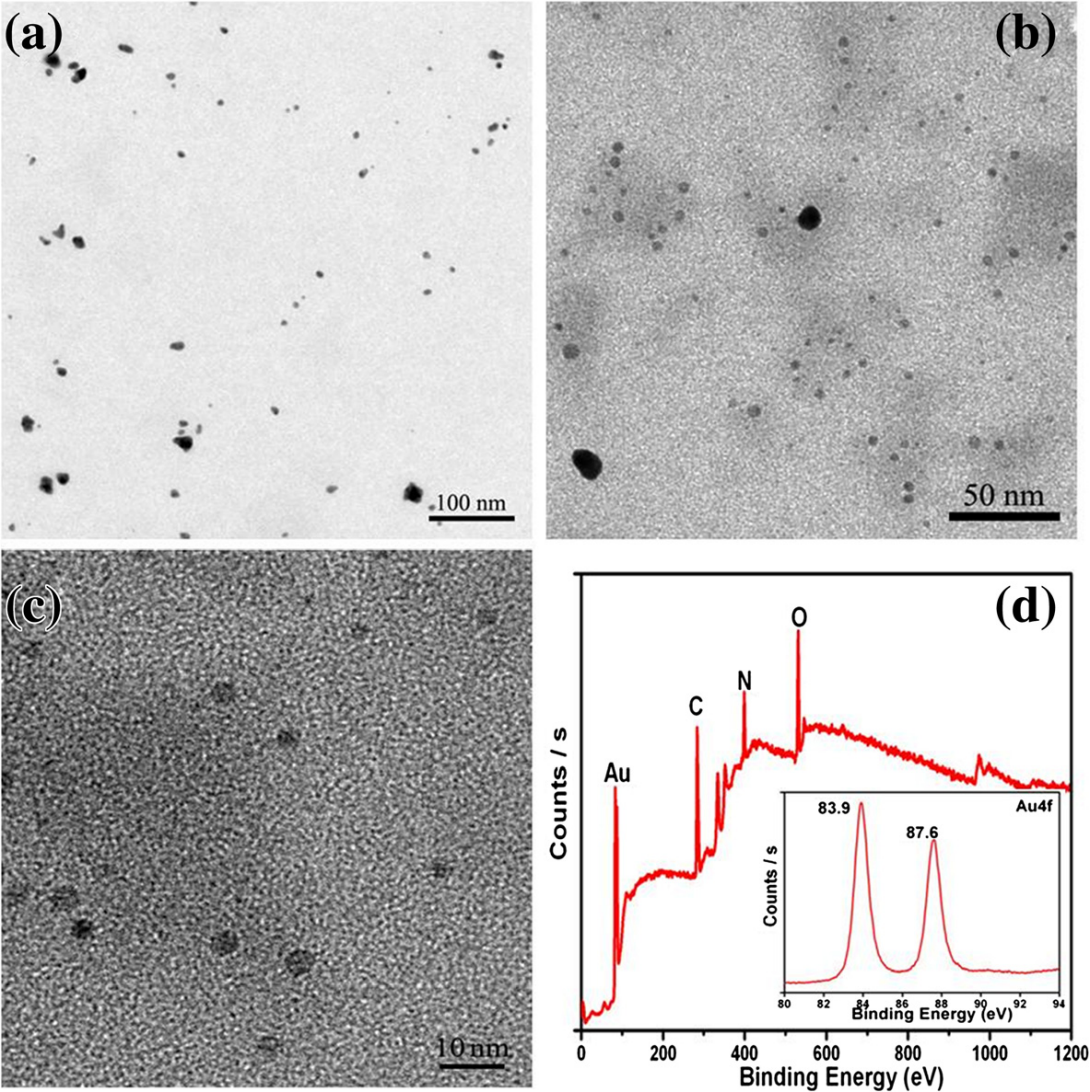
**TEM images and XPS spectrum. (a**, **b**, **c)** TEM images of BSA-Au nanocomplexes with different magnifications and **(d)** XPS spectrum of BSA-Au nanocomplexes; the inset is the XPS spectrum of the Au 4*f* band.

Transmission electron microscopy (TEM) images of BSA-Au nanocomplexes are shown in Figure 1a, b, c, which indicate that the nanocomplexes are spherical. In Figure 1b, c, the BSA-Au nanocomplexes show good dispersity. However, few particles tended to form aggregates (Figure 1a, b), which are attributed to the collision and fusion mechanism [20]. After the gold ions are reduced by N_2_H_4_·H_2_O, the newly generated ultrasmall nanoparticles have high surface activities, so the random collision is inevitable. Upon collision, these ultrasmall nanoparticles will fuse together by eliminating the high-energy surfaces with the increase of aging time [20]. In theory, the BSA molecules on the surface of the synthesized nanocomplexes, due to their low electron density, are not easy to observe by TEM microscopy. Interestingly, to the aggregates, the BSA layer is very clear and surrounds the surface of the aggregates (Additional file 1: Figure S1).

The X-ray photoelectron spectroscopy (XPS) spectrum (Figure 1d) shows the existence of C, N, O, and Au in the BSA-Au nanocomplexes. The peaks of C, N, and O elements are due to the presence of BSA. The inset spectrum of the Au 4*f* band confirms the presence of the Au element in the products. The FT-IR spectrum of the BSA-Au nanocomplex is similar to that of BSA (Additional file 1: Figure S2), which indicates that the BSA plays a direction role in the reaction progress.

**Figure 2:**
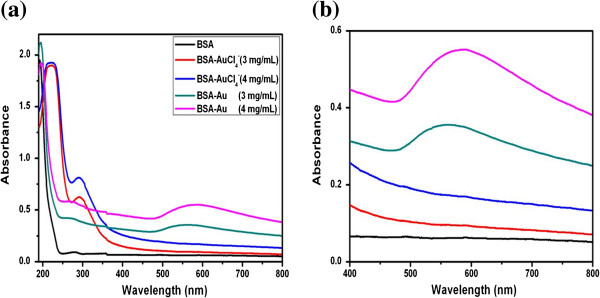
**UV–vis spectra of pure BSA, BSA-AuCl**_**4**_^**−**^**, and BSA-Au nanocomplexes. (a)** Low magnification and **(b)** high magnification.

Figure 2 shows the UV–vis spectra of pure BSA, BSA-AuCl_4_^−^, and BSA-Au nanocomplexes. The pure BSA has two characteristic absorption peaks at 192 and 280 nm; the former is assigned to the transition of P→P* of BSA’s characteristic polypeptide backbone structure C=O, and the latter is ascribed to the π→π* transition of the aromatic amino acid residues [10]. When the BSA-AuCl_4_^−^ complexes were formed, the two characteristic absorption peaks of BSA shift to 220 and 291 nm, respectively. Meanwhile, the intensity of the peak at 291 nm displayed a significant enhancement. These changes can be attributed to the chelation between AuCl_4_^−^ ions and BSA molecules and suggested that the conformation of the secondary structures of BSA had some changes. After the BSA-Au nanocomplexes were generated, the sites of two characteristic absorption peaks reverted to the original sites, which indicated that some groups were freed from the interaction between the AuCl_4_^−^ ions and BSA molecules. As shown in Figure 2b, the BSA-Au nanocomplexes exhibit a characteristic surface plasmon resonance band at approximately 556 and 585 nm, which corresponds to different BSA concentrations of 3 and 4 mg/mL, respectively.

**Figure 3:**
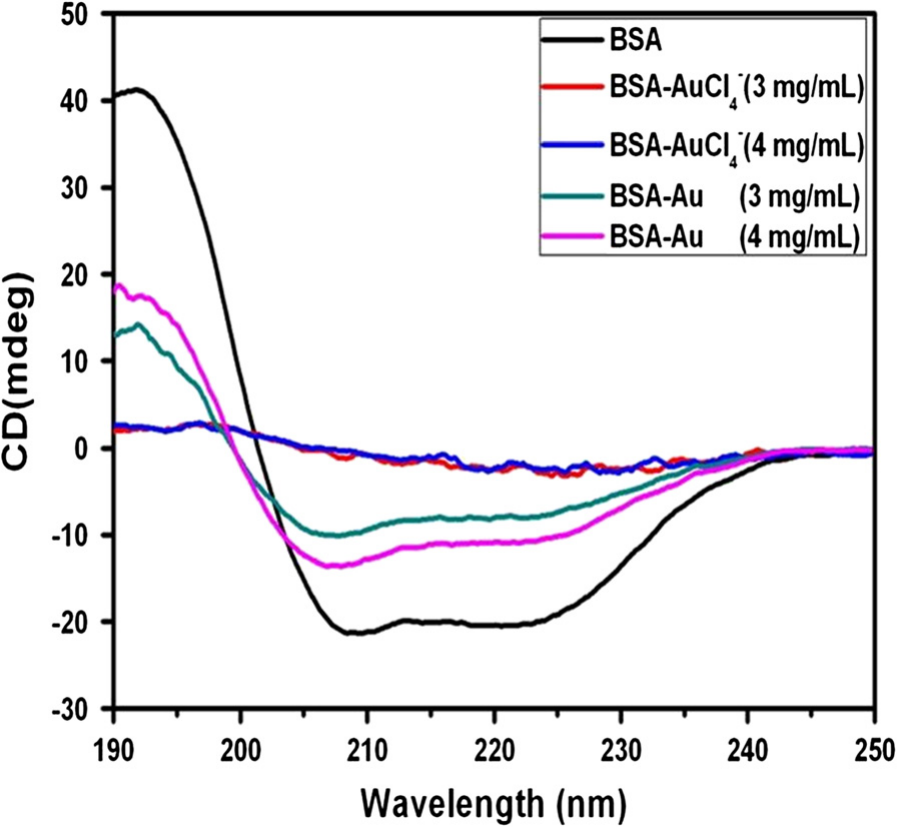
**CD spectra of pure BSA, BSA-AuCl**_**4**_^**−**^**, and BSA-Au nanocomplexes.**

The interaction between BSA and gold nanocomplexes has also been investigated using a circular dichroism (CD) spectropolarimeter. Figure 3 shows the CD spectra of pure BSA, BSA-AuCl_4_^−^, and BSA-Au nanocomplexes. The pure BSA showed a positive absorption band at 190 nm and two negative absorption bands at 209 and 222 nm [10]. When a certain amount of AuCl_4_^−^ was added into the pure BSA solutions, the bands at 190, 209, and 222 nm almost disappeared, which can be attributed to the strong chelation between the AuCl_4_^−^ ions and BSA molecules. The result indicated that the peptide chain in the α-helix structure of BSA extended and became a linear primary structure. Along with the extension of the peptide chain, more and more aromatic amino acid residues were exposed from the interior of BSA, so the changes were also very obvious in the UV spectra. After the formation of BSA-Au nanocomplexes, the positive peak at 190 nm ascended and the two negative peaks at 209 and 222 nm declined, which suggested that the conformation of the secondary structures of BSA was partially recuperative. The above results are in accord with the UV–vis spectra.

**Figure 4:**
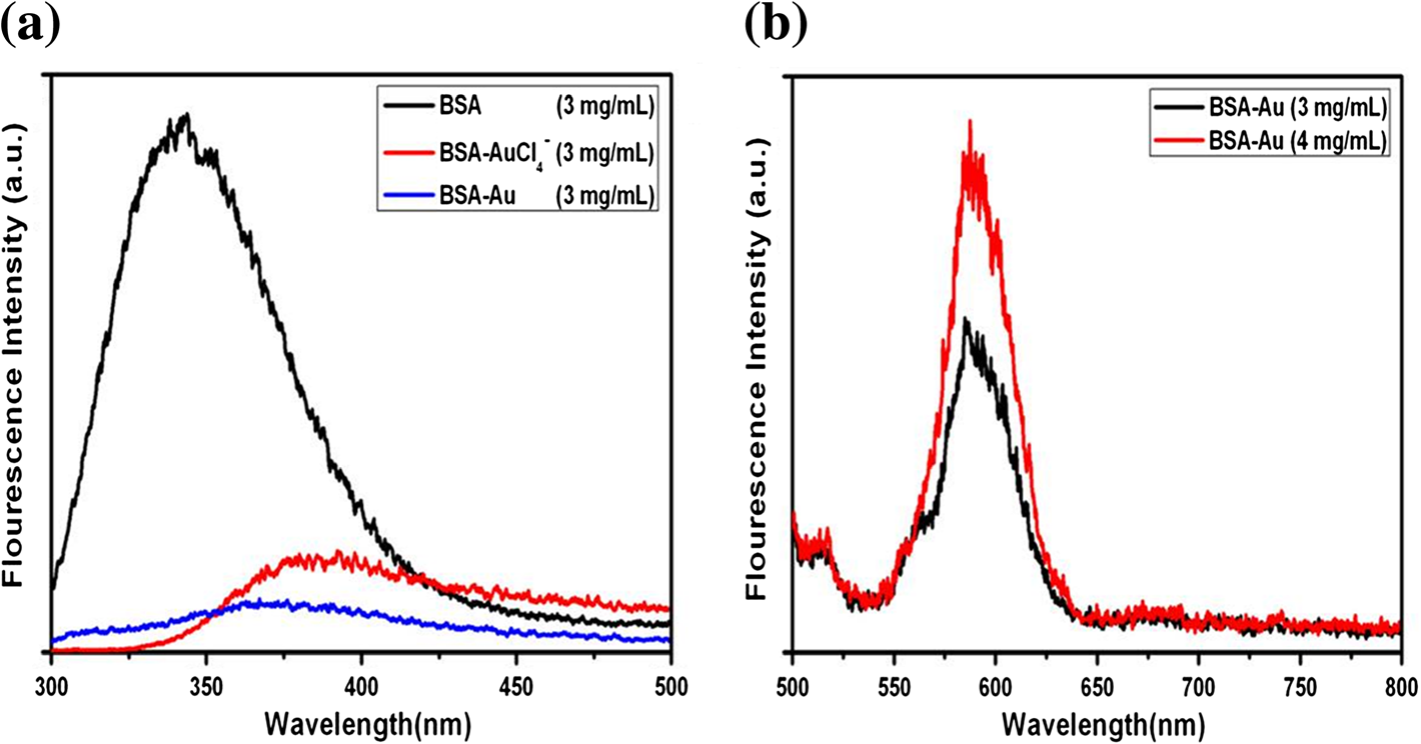
**Fluorescence emission spectra. (a)** Fluorescence emission spectra of tryptophan residues of pure BSA, BSA-AuCl_4_^−^, and BSA-Au nanocomplexes (*λ*_ex_ = 280 nm). **(b)** Fluorescence emission spectra of BSA-Au nanocomplexes in different concentrations of BSA solution (*λ*_ex_ = 470 nm).

To further investigate the interaction between BSA and gold nanocomplexes, fluorescence spectra were recorded on a Hitachih FL-4600 spectrofluorimeter (Hitachi Ltd., Tokyo, Japan). For protein with intrinsic fluorescence, more specific local information can be obtained by selectively exciting the tryptophan (Trp) residues. A BSA molecule possesses two Trp residues [21]. One is located on the bottom of hydrophobic pocket in domain II (Trp-213), while another is located on the surface of the molecule in domain I (Trp-134) [22]. Figure 4a shows the emission spectra of tryptophan residues of pure BSA, BSA-AuCl_4_^−^, and BSA-Au nanocomplexes. The choice of 280 nm as the excitation wavelength was to avoid the contribution from tyrosine residues. As shown, the fluorescence intensity was found to decrease with the addition of the AuCl_4_^−^ ions and the formation of gold nanocomplexes, while the emission maximum shifted from 350 to 380 nm (BSA-AuCl_4_^−^) and 370 nm (BSA-Au nanocomplexes). These different fluorescent characteristics actually reflected different conformational states of BSA, which agree with CD spectra. The results also indicated that there are strong interactions between the Trp residues of BSA and AuCl_4_^−^/gold nanocomplexes. The as-prepared BSA-Au nanocomplexes in different concentrations of BSA solution have a similar photoemission peak at approximately 588 nm, which implied that the nanocomplexes can be used as fluorescence probes for cell imaging.

**Figure 5:**
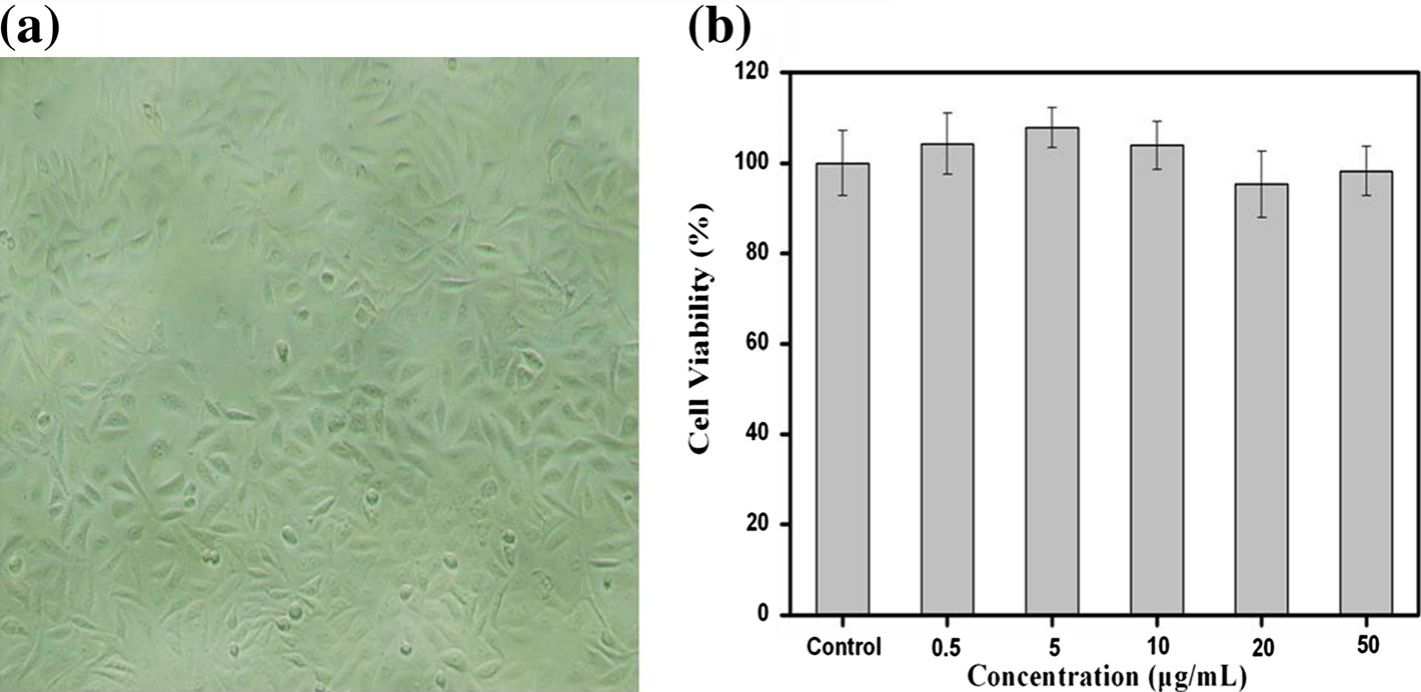
**Cytotoxicity of BSA-Au nanocomplexes on MGC803 cells. (a)** Morphology of MGC803 cells incubated with 50 μg/mL of BSA-Au nanocomplexes for 24 h at 37°C. **(b)** Dark toxicity of BSA-Au nanocomplexes to MGC803 cells incubated with 0~50 μg/mL of nanocomplexes for 24 h at 37°C. Cell viability was determined by MTT assay. Data represents mean ± SD (*n* = 5).

For further biomedical applications of BSA-Au nanocomplexes, cytotoxicity assessment on cells is essential to evaluate the potential. MTT assay was employed to investigate the cell viability of MGC803 cells incubated with different concentrations of BSA-Au nanocomplexes. Figure 5a shows that negligible cell death and physiological state change of MGC803 cells were observed, even if treated with the highest dosage (50 μg/mL) of BSA-Au nanocomplexes. Data obtained from MTT assay indicated no cytotoxicity of BSA-Au nanocomplexes in the concentration range of 0~50 μg/mL, cell viability are more than 95% in comparison with control group (Figure 5b). These results indicated that BSA-Au nanocomplexes possessed non-cytotoxicity and excellent biocompatibility on MGC803 cells within 0~50 μg/mL.

BSA, a ubiquitous plasma protein with a molecular weight of 66,500 Da, is composed of 580 amino acid residues [23,24]. Due to their wide hydrophobic, hydrophilic, anionic, and cationic properties, BSA has been extensively used as a model protein in many fields including drug delivery [25], biomimetic mineralization [26], nanomaterial synthesis [27,28], surface modification and intermolecular interaction [29], etc. More recently, our group has successfully prepared a series of semiconductor chalcogenides with different sizes and morphologies in a solution of BSA at room temperature [10,27,30]. In this case, BSA plays multifunctional roles: (1) to direct the synthesis of Au nanocomplexes, (2) to stabilize the Au nanocomplexes, (3) to improve the biocompatibility of Au nanocomplexes, and (4) to provide bioactive functionalities into these nanocomplexes for further biological interactions or coupling.

**Figure 6:**
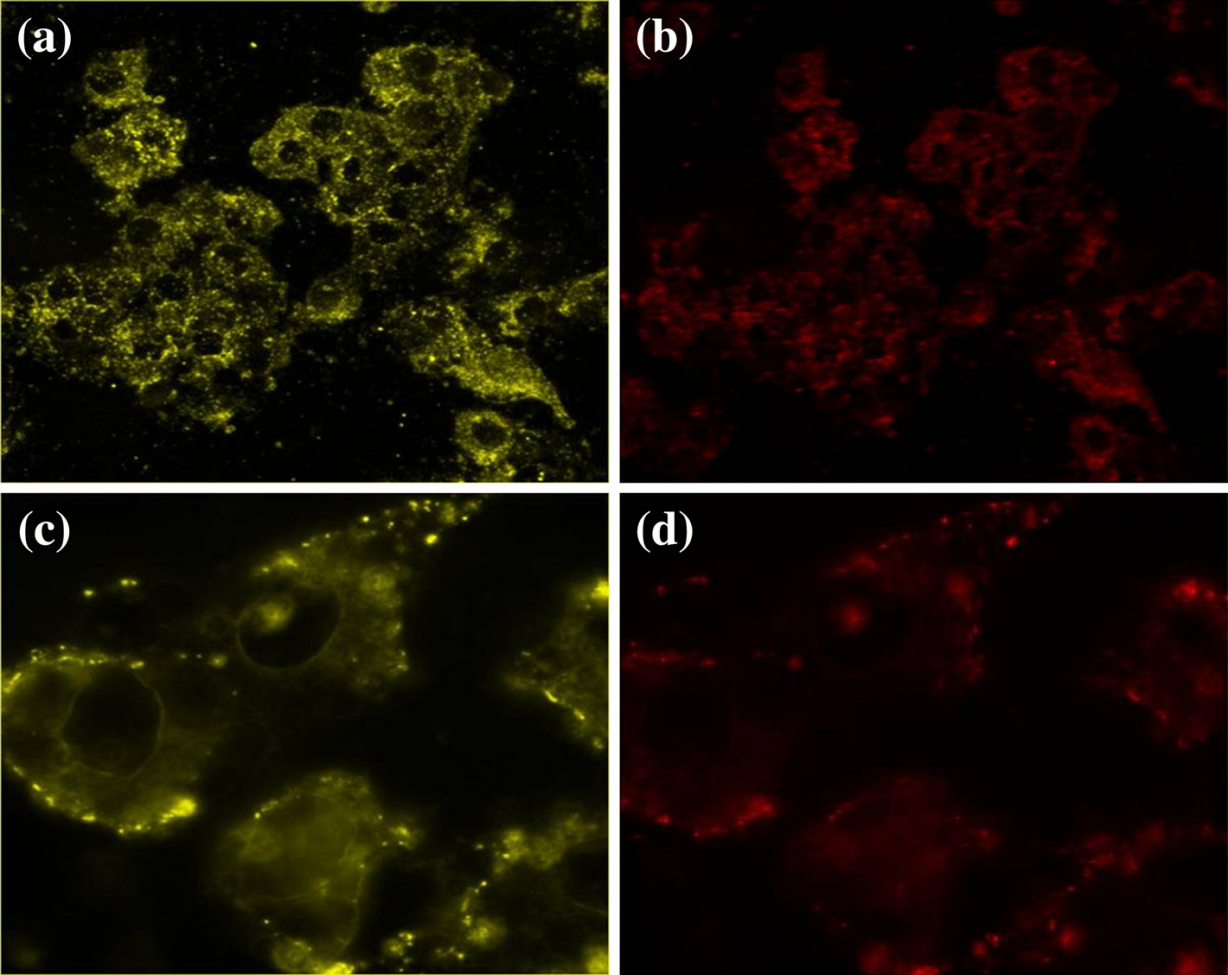
**Dark-field scattering images (a, c) and fluorescence images (b, d). (a**, **b)** Low-magnification image of targeted MGC803 cells incubated with 50 μg/mL of BSA-Au nanocomplexes for 2 h. **(c**, **d)** High-magnification image of targeted MGC803 cells incubated with 50 μg/mL of BSA-Au nanocomplexes for 30 min, monitored by dark-field and fluorescence microscopy.

An appropriate use of such nanocomplexes for biological labeling requires the decoration of biomarker molecules on the nanocomplexes’ surface [31,32]. Folic acid (FA) molecules, actively targeting the folate receptors of cancer cells, were selected as a model and conjugated with BSA-Au-NH_2_ using a modification of the standard EDC-NHS reaction as described by Jönsson [33-35]. To determine the intracellular uptake and the targeting ability of BSA-Au-FA, dark-field scattering and fluorescence imaging were performed on MGC803 cells (Figure 6). At 2 h after being incubated with 50 μg/mL of BSA-Au-FA, cells displayed an intense homogeneous cytoplasmic golden color (Figure 6a) and an intense homogeneous cytoplasmic red color (Figure 6b) around the nucleus, indicating accumulation of BSA-Au-FA nanocomplexes in cells. With regard to the targeting ability of BSA-Au-FA, we evaluated the cellular selective uptake of BSA-Au-FA with a MGC803 cell in an RPMI-1640 medium without FA, which was carried out and contrasted with the other two groups: (a) cells treated with BSA-Au in RPMI-1640 medium without FA and (b) cells treated with BSA-Au-FA in RPMI-1640 medium with FA. After a 30-min incubation, only the cells incubated with BSA-Au-FA in RPMI-1640 medium without FA displayed abundant golden dots (Figure 6c) and a red fluorescence signal (Figure 6d) on the membrane of the cells, indicating selective targeting of nanocomplexes on MGC803 cells.

## Conclusion

In summary, biocompatible BSA-Au nanocomplexes were successfully synthesized in water at room temperature by a protein-directed, solution-phase, green synthesis method. The as-prepared BSA-Au nanocomplexes showed highly selective targeting and dark-field and fluorescence imaging on MGC803 cells. It may have great potential in applications such as tumor targeting imaging, drug delivery, and ultrasensitive detection. The current study provides further evidence of the biomimetic fabrication of functional materials and exemplifies the interactions between proteins and metal nanomaterials in an attempt to create novel bioconjugated composites.

## Competing interests

The authors declare that they have no competing interests.

## Authors’ contributions

JL designed and performed all the experiments and wrote the manuscript. ZZ helped prepare the gold nanoclusters/nanoparticles. ZL, CZ, and XW contributed to cell imaging. KW finished the MTT assay. GG and PH participated in the design of the study and discussion. DC conceived the study and participated in its design and coordination. All authors read and approved the final manuscript.

## Supplementary Material

Additional file 1 **Supporting information.** A document showing two supplementary figures: the TEM image of BSA-Au nanocomplexes in long aging time and the FT-IR spectra of (a) BSA and (b) BSA-Au nanocomplexes. Click here for file
