# Supplementary material for: Biomimetic one-pot synthesis of gold nanoclusters/nanoparticles for targeted tumor cellular dual-modality imaging
Source: Nanoscale Res Lett. 2013 Apr 15;8(1):170. doi: 10.1186/1556-276X-8-170 (PMC3637621; doi:10.1186/1556-276X-8-170)
Supplement: Additional file 1 — Supporting information. A document showing two supplementary figures: the TEM image of BSA-Au nanocomplexes in long aging time and the FT-IR spectra of (a) BSA and (b) BSA-Au nanocomplexes. [file 1556-276X-8-170-S1.doc]

**Supporting Information**


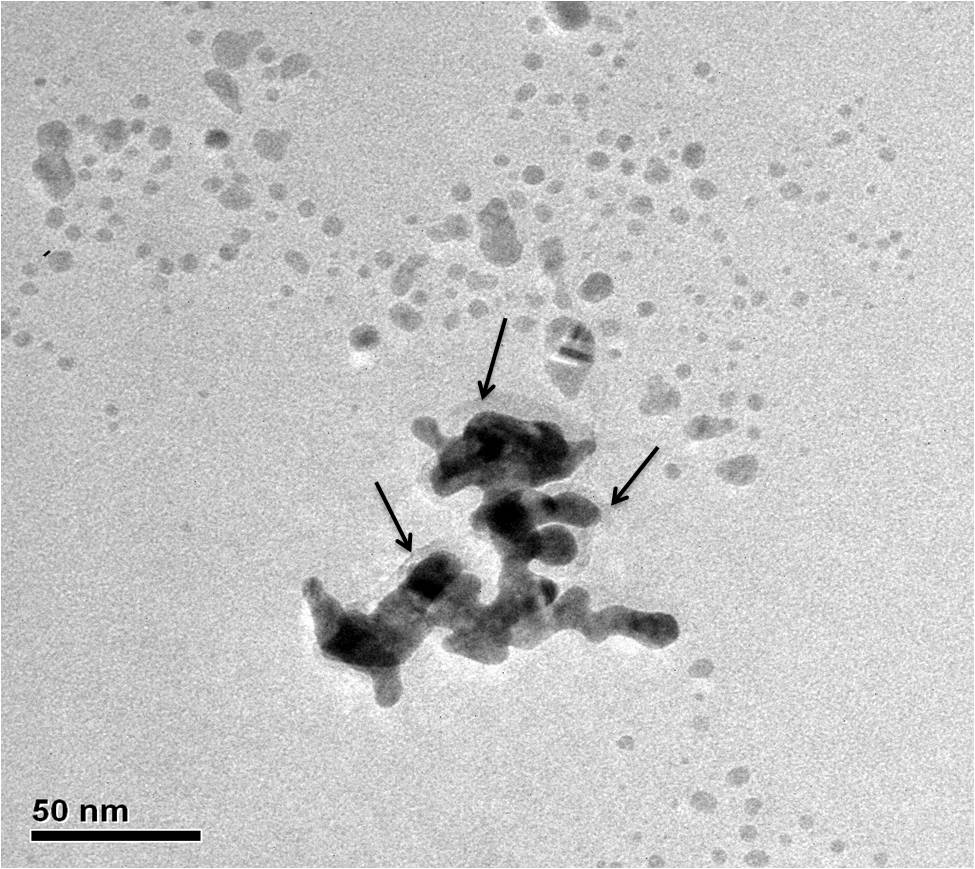


**Fig. S1** TEM image of BSA-Au nanocomplexes in long aging time, Arrows denote the BSA layers.


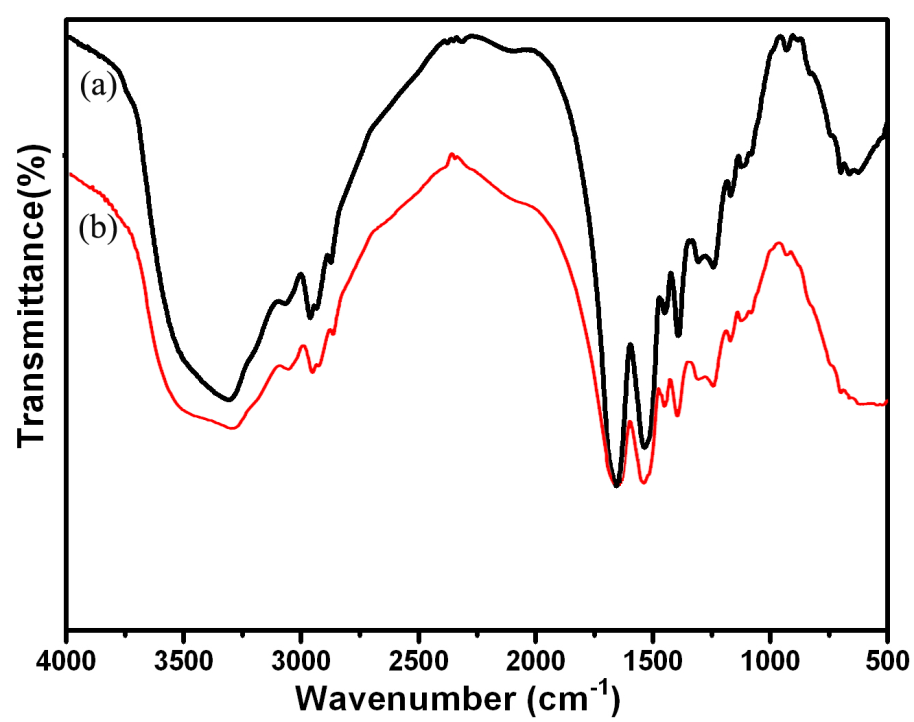


**Fig. S2** FT-IR spectra of (a) BSA, and (b) BSA-Au nanocomplexes.
